# Supplementary material for: sCNAphase: using haplotype resolved read depth to genotype somatic copy number alterations from low cellularity aneuploid tumors
Source: Nucleic Acids Res. 2016 Nov 28;45(5):e34. doi: 10.1093/nar/gkw1086 (PMC5389684; doi:10.1093/nar/gkw1086)
Supplement: Supplementary Data [file gkw1086_supplementary_data.zip › nar-02000-met-n-2016-File002.pdf]

Supplementary Tables

Supplementary Table S1. A list of tools designed for copy number profile for impure tumor samples.

Tool	TC	Ploidy parameter	Paired Normal	GC-bias corr.	LOH	Data type	Model-free pre-segmentation	Reference s
**Absolute**	Y	Y	Y	-	Y	Array NGS	Based on HAPSEG	(1,2)
**ASCAT**	Y	Y	Opt	Opt	Y	Array NGS	Itself	(3)
**PICNIC**	N	Y				Array	No	
**OncoSNP**	Y	N	Y	Y	Y	Array	No	(4)
**PyLOH**	Y	N	Y	Y	Y	NGS	BIC-seq	(5,6)
**CLImAT**	Y	N	N	Y	Y	NGS	No	(7)
**THetA2**	Y	N	Y	Y	Y	NGS	BIC-seq	(8)
**OncoSNP-Seq**	Y	N	Y	Y	Y	NGS	No	(9)
**CNAnorm**	Y	Y	Y	Y	N	NGS	No	(10)

Supplementary Table S2. The source of the mixture samples.

Tumor purity	HCC1143 (TCGA)	HCC1954 (TCGA)	HCC1187 (Illumina)	HCC2218 (Illumina)
**100%**	Downloaded	Downloaded	Downloaded	Downloaded
**95%**	Downloaded	Downloaded	Generated in-house	Generated in-house
**80%**	Downloaded	Downloaded	Generated in-house	Generated in-house
**60%**	Downloaded	Downloaded	Generated in-house	Generated in-house
**40%**	Downloaded	Downloaded	Generated in-house	Generated in-house
**20%**	Downloaded	Downloaded	Generated in-house	Generated in-house
**10%**	Generated in-house	Generated in-house	Generated in-house	Generated in-house
**5%**	Downloaded	Downloaded	Generated in-house	Generated in-house
**0%**	Downloaded	Downloaded	Generated in-house	Generated in-house

Supplementary Table S3. The estimated tumor purity, tumor cellularity, tumor ploidy for tumor-normal mixtures.

	HCC1143 (Hypo-tetraploid)										
	sCNAphase			CLImAT				ASCAT			
TP	*tc(%)*	*tp(%)*	*pl*		*tc(%)*	*tp(%)*	*Pl*		*tc(%)*	*tp(%)*	*pl*
100%	100	100	3.7		98	99	3.77		1.00	99	3.82
95%	91	95	3.7		82	90	3.80		0.93	90	3.80
80%	68	80	3.7		65	78	3.80		0.68	78	3.78
60%	45	60	3.8		43	60	3.91		0.44	60	3.77
40%	27	40	3.8		33	43	3.03		0.27	43	3.76
20%	12	21	3.8		19	27	3.19		0.31	27	1.87
10%	6	11	3.7		48	50	2.19		0.28	50	2.03
5%	4	7	3.7		71	73	2.16		0.29	73	2.00
0%	0.01	0.01	1.8		60	61	2.16		0.27	62	2.00
	**HCC1187 (Hypo-triploid)**										
100%	100	100	2.8		89	92	2.68		1.00	92	2.72
95%	93	95	2.7		85	88	2.70		0.93	88	2.72
80%	74	80	2.7		71	77	2.69		0.74	77	2.72
60%	52	60	2.7		52	59	2.70		0.51	59	2.73
40%	33	40	2.7		32	39	2.71		0.32	39	2.73
20%	19	23	2.7		18	23	2.75		0.21	23	2.6
10%	8	11	2.7		58	58	2.02		NA	58	NA
5%	4	5	2.8		62	62	2.02		0.22	62	2.00
0%	0.01	0.01	2.5		65	65	2.01		0.25	65	1.99
	**HCC1954 (Hyper-tetraploid)**										
100%	100	1.00	4.5		49	56	2.67		1.00	56	4.6
95%	86	93	4.7		46	54	2.72		0.97	54	4.2
80%	60	78	4.7		41	48	2.62		0.58	48	4.8
60%	37	58	4.7		36	41	2.50		0.4	41	4.6
40%	22	39	4.7		20	27	2.98		0.24	27	4.5
20%	11	21	4.7		51	52	2.09		0.24	52	2.2
10%	6	12	4.9		50	51	2.05		0.25	51	2.07
5%	4	6	2.8		52	52	1.99		0.26	52	2.02
0%	0.01	0.01	2.3		64	65	2.16		0.26	66	200
	**HCC2218 (Tetraploid)**										
100%	100	100	4.2		48	51	2.3		0.99	51	4.32
95%	89	95	4.2		46	49	2.29		0.9	49	4.36
80%	64	79	4.2		76	77	2.14		0.64	77	4.32
60%	40	59	4.2		54	56	2.16		0.4	56	4.33
40%	23	39	4.4		34	35	2.12		0.4	35	2.08
20%	11	21	4.4		22	23	2.12		0.23	23	2.12
10%	5	11	4.4		41	41	2.02		0.25	41	2.06
5%	5	5	2.2		43	43	2.01		0.23	43	2.01
0%	0.0	0.04	2.4		45	44	1.98		0.24	45	1.99

In the table, *tc* denotes for tumor cellularity, pl for ploidy and *tp* for tumor purity. The ploidy for HCC1143, HCC1954 and HCC1187 were derived from the SKY data (http://old-

www.path.cam.ac.uk/~pawefish/BreastCellLineDescriptions/HCC1143.html). The ploidy of HCC2218 is found from COSMIC (http://grch37-cancer.sanger.ac.uk/cosmic/sample/overview?id=749716).

Supplementary Table S4. The capacity of CLImAT to detect the focal sCNAs in COSMIC.

Tumor purity	HCC1143 15 focal amplification from COSMIC		HCC1954 94 focal amplification from COSMIC		HCC1187 2 focal amplification from COSMIC		HCC2218 18 focal amplification from COSMIC	
	Sen	Spe	Sen	Spe	Sen	Spe	Sen	Spe
**100%**	33	17	44	55	50	25	6	33
**80%**	40	17	39	51	50	8	0	0
**60%**	47	13	14	36	100	13	0	0
**40%**	20	40	35	39	100	9	22	50
**20%**	53	31	0	0	100	6	22	25
**10%**	0	0	0	0	0	0	0	0

Sen for sensitivity; Spe for specificity.

Supplementary Table S5. The detection of ERBB2 and chr11q13 amplification across the mixtures.

	**ERBB2**		**chr11q13**	
TP	*HCC1954*	*HCC2218*	*HCC1143*	*HCC1954*
100%	>=12	>=12	>=12	>=12
95%	>=12	>=12	>=12	>=12
80%	>=12	>=12	>=12	>=12
60%	>=12	>=12	>=12	>=12
40%	>=12	>=12	>=12	>=12
20%	>=12	>=12	>=12	>=12
10%	>=12	>=12	>=12	>=12
5%	>=12	>=12	>=12	>=12

Supplementary Table S6. Average 50% overlap specificity and sensitivity across four cell lines as a function of sCNAphase detection threshold.

Tumor purity	sCNAphase threshold = 2*ploidy		sCNAphase threshold = 2*ploidy + 1		sCNAphase threshold = 2*ploidy + 2	
	**Sen**	**Spe**	**Sen**	**Spe**	**Sen**	**Spe**
**100%**	0.67	0.41	0.58	0.59	0.51	0.70
**80%**	0.62	0.39	0.54	0.58	0.50	0.72
**60%**	0.67	0.39	0.60	0.58	0.53	0.72
**40%**	0.63	0.41	0.56	0.59	0.50	0.75
**20%**	0.64	0.42	0.60	0.57	0.53	0.72
**10%**	0.67	0.47	0.59	0.64	0.48	0.75
**5%**	0.41	0.62	0.32	0.69	0.27	0.79

Supplementary Table S7. A list of the homozygous deletions greater than 100kb in the COSMIC data.

Cell-Line	Location	Size	Detected by sCNAphase	Detected by CLImAT
**HCC1143**	Chr4:92,308,295-92,882,506	574 kb	10% Mixture	Not detected
**HCC1187**	Chr6:134,329,397-134,537,448	208 kb	Not detected	Not detected
**HCC1187**	Chr14:63,703,884-64,334,878	630 kb	10% Mixture	Not detected
**HCC1187**	Chr15:72,194,262-72,443,790	249 kb	10% Mixture	Not detected
**HCC2218**	Chr16:68,698,153-68,844,967	146 kb	Not detected	Not detected
**HCC1954**	Chr9:45,706,445-46,038,862	332 kb	Not detected	Not detected
**HCC1954**	Chr11:46,535,802-46,636,287	100 kb	Not detected	Not detected
**HCC1954**	Chr22:16,239,488-16,395,163	155 kb	Not detected	Not detected

Supplementary Table S8. Consensus detection of TP53 LOH at different levels of tumor purity.

	Location of TP3 (chr17:7,571,720-7,590,868)	
**Cell-Line**	Detected LOHs by sCNAphase	Detected LOHs by CLImAT
**HCC1143**	100%-20% Mixture (4 copies)<br>5% mixture (4 copies)	100%-20% Mixture
**HCC1187**	100%-5% Mixture (2 copies)	100%-20% Mixture
**HCC2218**	100%-10% Mixture (2 copies)<br>5% Mixture (1 copy)	80%-20% Mixture
**HCC1954**	100% Mixture (4 copies)<br>95% Mixture (4 copies)<br>80-40% Mixture (4 copies)<br>20%-10% Mixture (3 copies)<br>5% Mixture (1 copy)	80%-60% Mixture

Supplementary Table S9. Germline Heterozygous SNPs present in in the TP53 loci.

Position	Ref	Alt	Depth	AF	Phase	Location	h0	h1	h0	Effect
							Normal		Tumor	
**chr17:7574775**	C	T	31	0.54	01	Non-coding	C	T	C	
**chr17:7574779**	C	T	30	0.53	01	Non-coding	C	T	C	
**chr17:7574936**	C	T	41	0.41	01	Non-coding	C	T	C	
**chr17:7577091**	G	A	29	0.55	--	CDS	A	G	A	R283C*
**chr17:7577644**	C	G	36	0.47	10	Non-coding	G	C	G	
**chr17:7578115**	T	C	34	0.5	10	Non-coding	C	T	C	
**chr17:7578645**	C	T	29	0.55	10	Non-coding	T	C	T	
**chr17:7578837**	A	G	38	0.28	10	Non-coding	G	A	G	
**chr17:7579472**	G	C	37	0.59	10	CDS	C	G	C	P72R
**chr17:7579801**	G	C	31	0.35	10	Non-coding	C	G	C	

Ref and alt are short for reference and alternative allele. AF is for allelic frequency in the normal sample, HCC2218BL. H0 and H1 stand for the two parental chromosomes. The 01 in the phase column means that the reference allele is from H0, the alternative allele is from H1. The 10 means the opposite.

Supplementary Figure

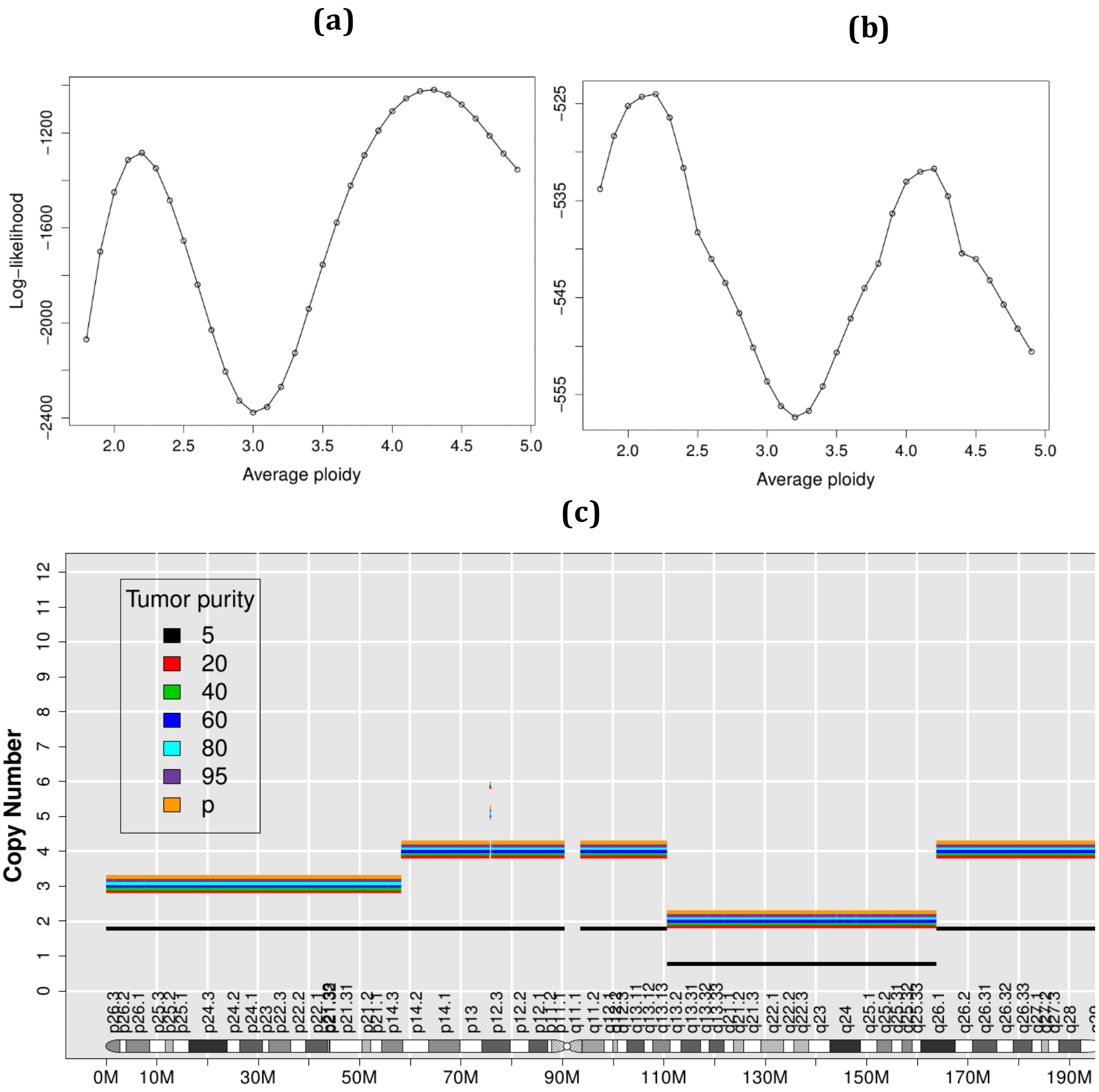

Supplementary Figure S1. Ploidy estimation at different tumor purity for HCC2218. As sCNAphase searches possible average ploidy values, (a) shows the maximum likelihood calculated for the ploidy of 1.9 to 5 for 100% tumor purity sample. There are two peaks corresponding to two possible ploidy estimation roughly at 2.3 or 4.3. In **Supplementary Figure** 4, the dSKY of HCC2218 shows the number of the chromosome arms were mostly estimated to be even numbers, except for a region in chr3p estimated to be 3 copy. Because this region supported for average ploidy of ~4.3, the peak at 4.3 is clearly higher, a preferred ploidy estimation. However, at the 5% tumor purity, the lower tumor ploidy estimation became more likely in (b). As shown in the copy number profile of chr3 HCC2218 in (c), this copy number reduced by a half at 5% tumor purity. Even though the reduction from 3 to 2 copies is unfavorable in region from 0 to 50M, this region cannot produce as strong support for the higher ploidy as for the pure tumor sample. In (c) legend, p stands for pure tumor samples.

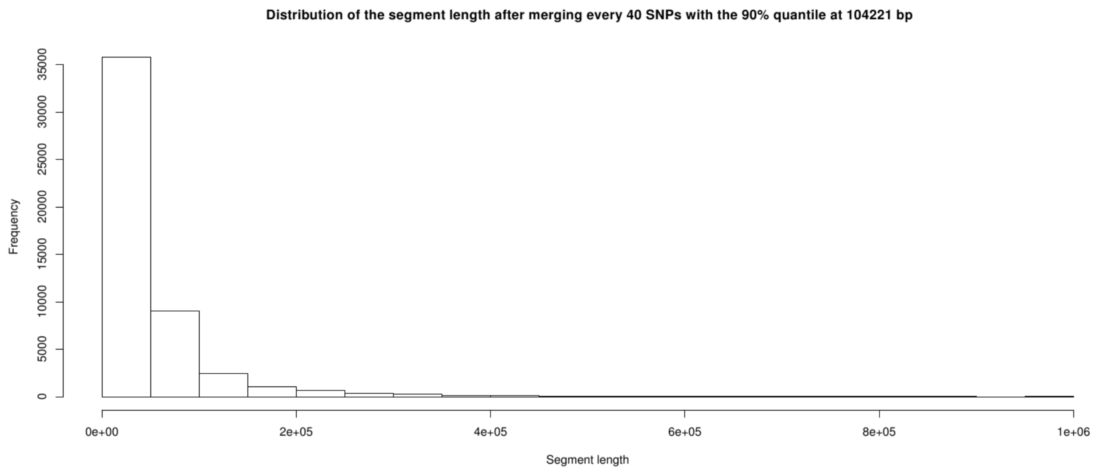

Supplementary Figure S2. The segment size distribution merging every 40 SNPs calculated from HCC1143.

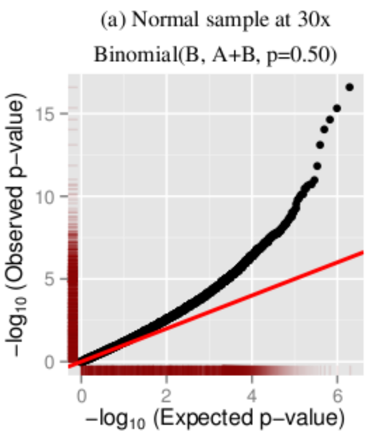

Supplementary Figure S3. Inflation of test-statistic for detecting somatic copy number aberation for normal sample under null assumption of BAF = 0.5**.** Each of the black dots in the Q-Q plots corresponded to a P-value for a pair of allelic depths under different assumptions. P-values were calculated under the assumption that both alleles had equal copy numbers for a normal samples. The red line, y=x, is the expected fitness under null hypothesis.

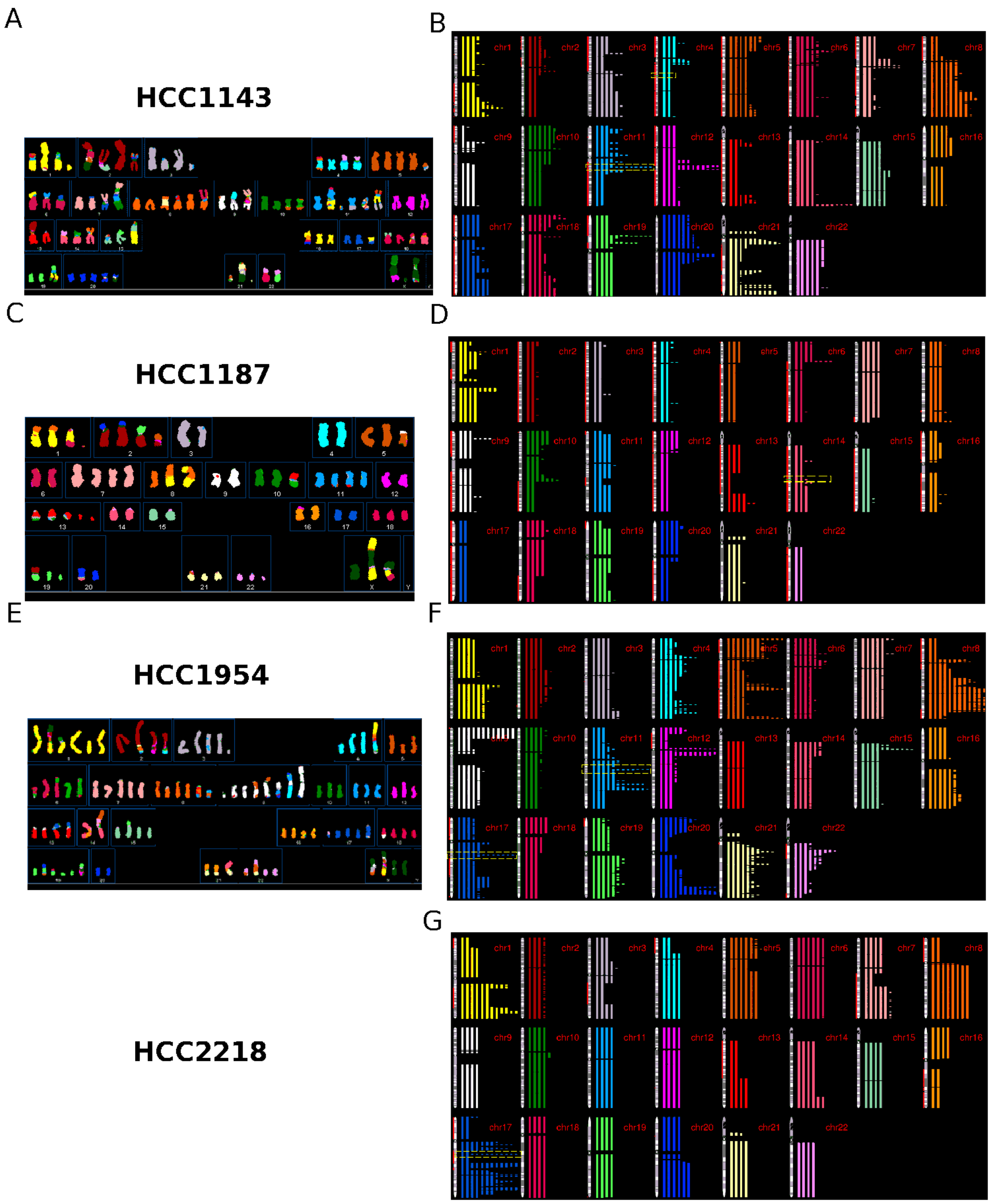

Supplementary Figure S4. dSKY plots capture the same large scale copy number change present in existing SKY experiments. (A), (C) and (E) all show the results from a traditional SKY analysis of the cell-lines characterised in this study. Each chromosome is represented by a specific colour and are grouped together, making it possible to count the number of copies of each chromosome. This work was completed by Mira Grigorova & Paul A.W. Edwards (Department of Pathology, University of Cambridge) - see http://www.path.cam.ac.uk/~pawefish - and is reproduced with permission. We were unable to find publicly available results from a SKY analysis of HCC2218. To allow a direct comparison between the result produced by sCNAphase and the SKY experiments, each chromosome was painted in the same colour used in the traditional SKY analysis and are presented in a similar layout. (B),(D),(F) and (G) all show the corresponding dSKY plots. To show additional information, such as the location of each sCNA and the regions that have undergone an LOH event, a diagram of each chromosome is present in the dSKY plot. This diagram is coloured red to indicate an LOH event or green to highlight a homozygous deletion. High resolution dSKY plots can be found from figshare at https://figshare.com/authors/_/1365237.

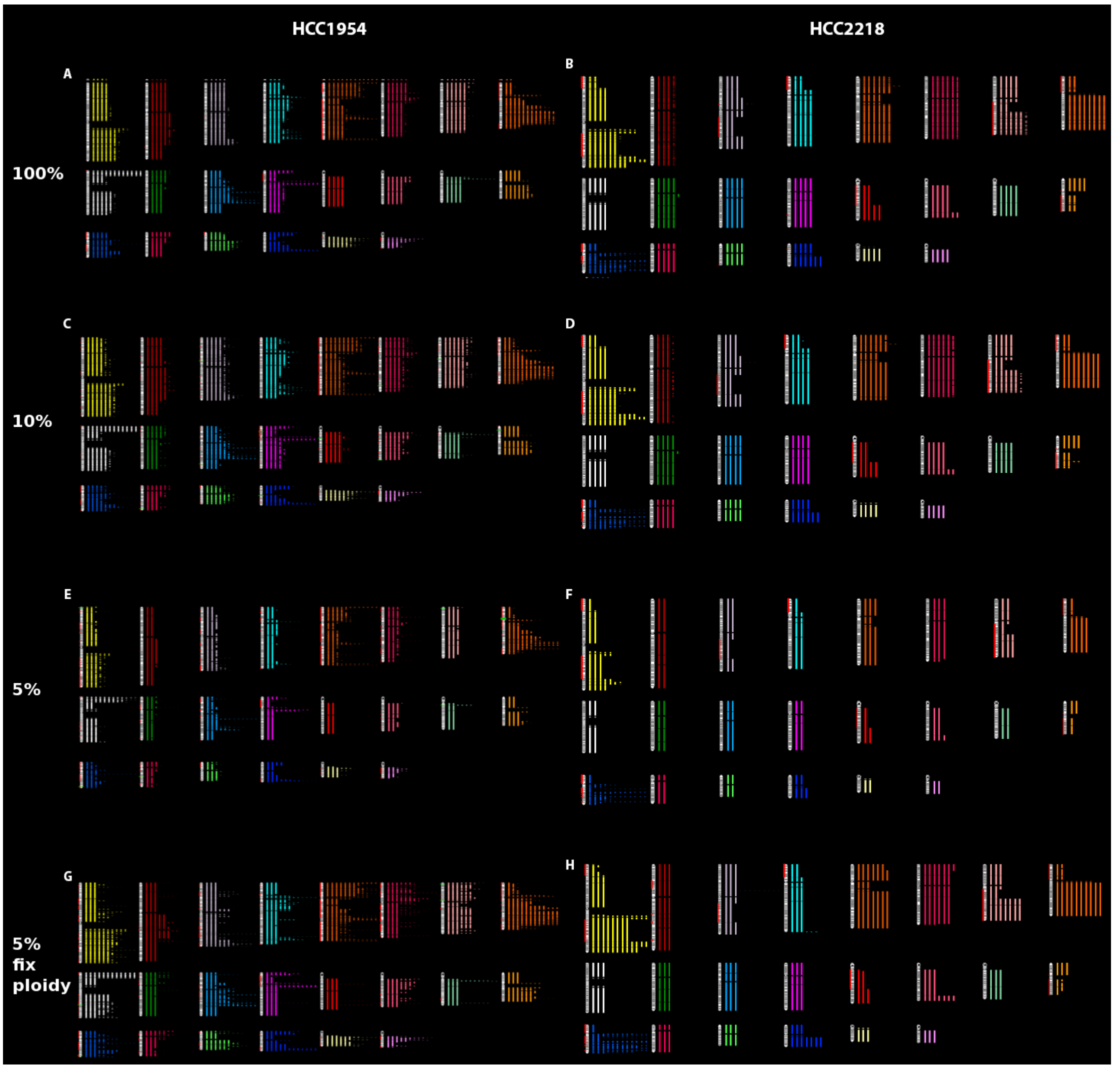

Supplementary Figure S5. Accurate copy number profiling relies on accurate determination of ploidy. Both sCNAphase and the independent SKY analysis of the pure HCC1954 and HCC2218 cell-lines classified them as tetraploids (A),(B), a result maintained across the majority of the mixtures, including those that only contained 20% of cell-line derived reads (C) and (D). However, in the 5% cell-line DNA mixtures, ploidy was miscalculated as a result, sCNAphase failed to produce an accurate copy number profile for these mixtures (E) and (F). When the incorrect plodiy value was manually replaced with the correct one, it was possible to identify the same broad copy number profile in the 5% mixture, that was present in the 100% cell-line mixture (G) and (H).

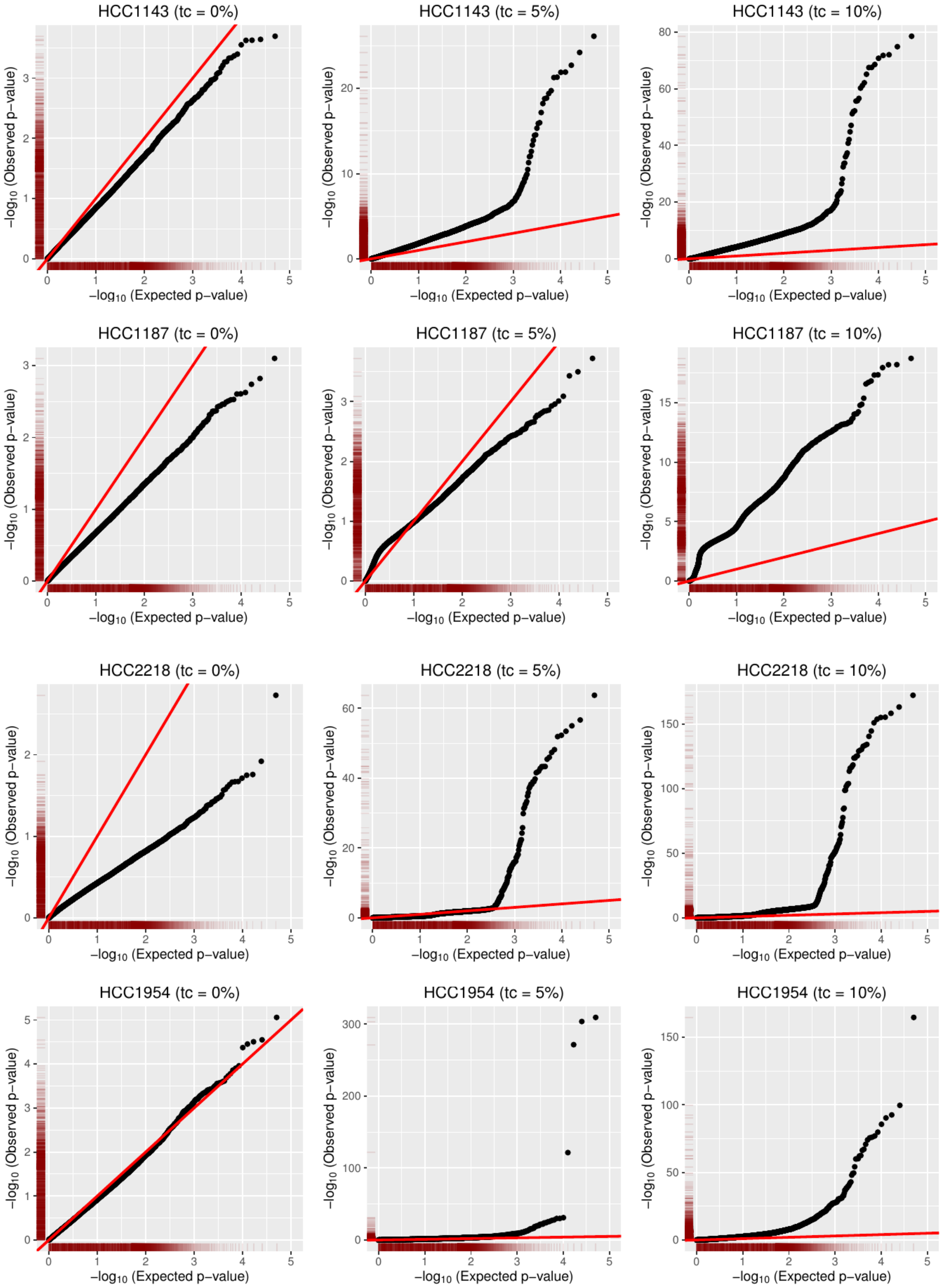

Supplementary Figure S6. Detection of the presence of tumor DNA in a mixture. This shows the quantile-quantile plot calculated from a null model assuming there is no tumor DNA present in the mixture for each of the 4 samples studied this paper (shown in rows) at different mixtures of tumour and normal DNA of 0%, 5% and 10% (columns). The red straight line is the baseline (expected distribution under the assumption). Inflation above the red line indicates the presence of tumor DNA. The presence of tumor DNA can be inferred for all 10% mixtures, and 3 of 4 5% mixtures, but not for any of the 0% mixtures.

A

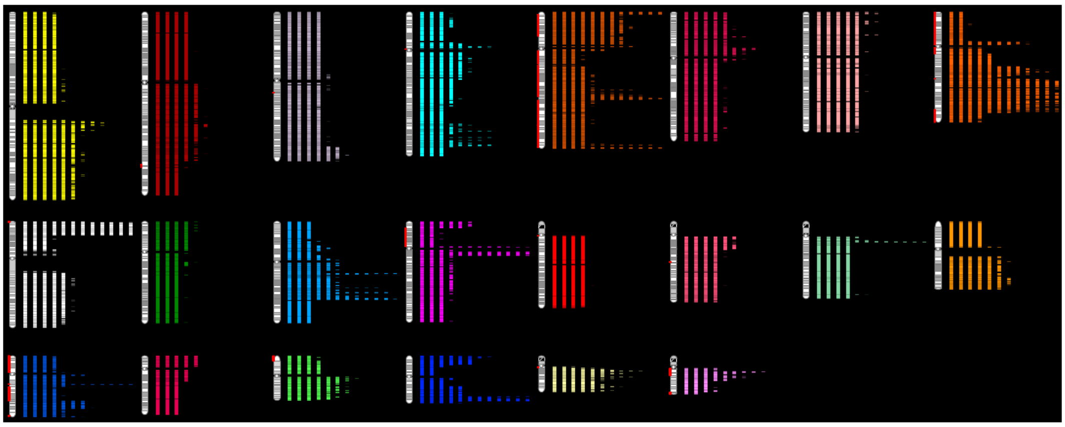

B

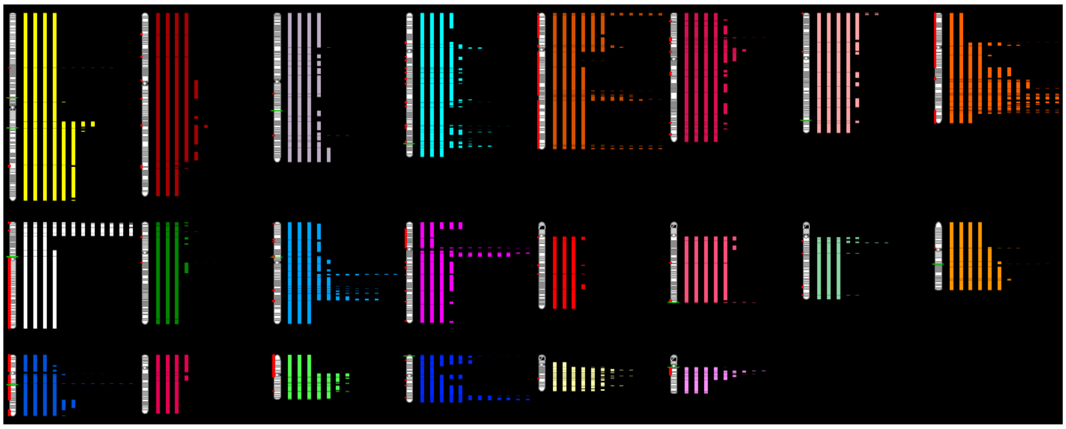

C

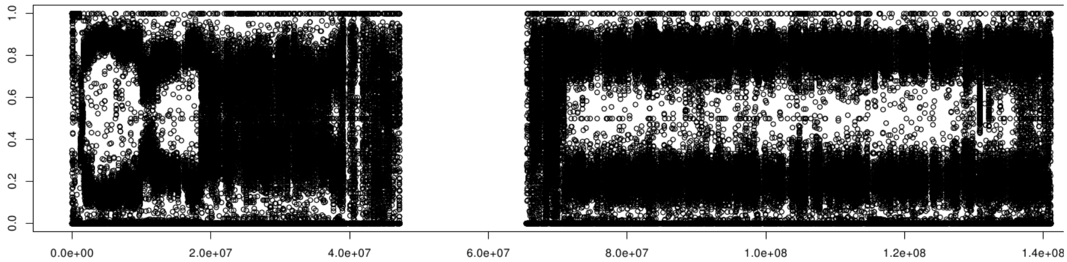

Supplementary Figure S7. Discrepancy about LOHs estimation for HCC1954 is presented in dSKY plots in (A) and (B) generated from sCNAphase and COSMIC respectively. The clear differences were found at chr9 q-arm, which is found to be LOHs only by COSMIC. The absolute copy number was predicted to be 4 from COSMIC than 5 from sCNAphase. However the B-allelic frequency at chr9q in (C) shows the allelic copy numbers at chr9 are very imbalance, allelic copy numbers of 4 to 1 from sCNAphase, but clearly the region remains heterozygous. Similarly the extra regions of LOHs from COSMIC at chr8, chr17 and chr19 are also likely to be heterozygous, as supported by the BAFs. These are also coincide with sight lower copy number estimation sCNAphase. COSMIC segmentation also under-estimated ploidy for chr7, chr15, which are 5 and 4 copies according to sCNAphase and the SKY plots (**Supplementary Figure S4E**) .

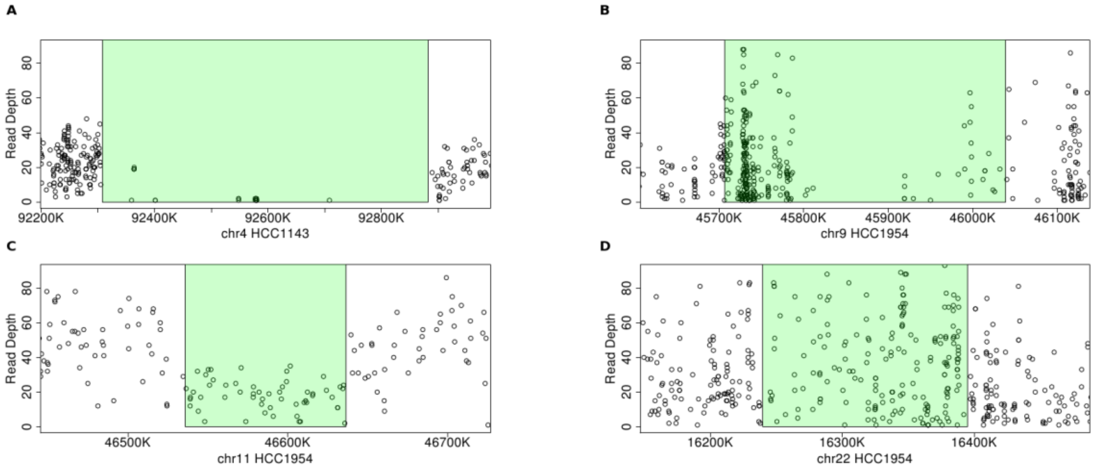

Supplementary Figure S8. The B-allelic frequency at regions with heterozygous deletions from COSMIC. The plots corresponds to the regions containing the homozygous deletions in HCC1143 and HCC1954 as shown in Supplementary Table 6. The green shading represents the location of the homozygous deletion listed in the COSMIC annotation of these cell-lines, while the black points present the read depth for the individual heterozygous SNPs located within these regions. For HCC1143 (A, C), there is a high level of consistency between deletion in the COSMIC annotation of this cell-line and the read depth of germ-line SNPs in the NGS data. In contrast, the results from HCC1954(B, D) do not show the same level of consistency. In the deletion on chromosome 9(B), the NGS data suggests that the deletion here is significantly smaller than the one listed by COSMIC. In contrast, the deletion on chromosome 22(D), is not consistent with the NGS data.

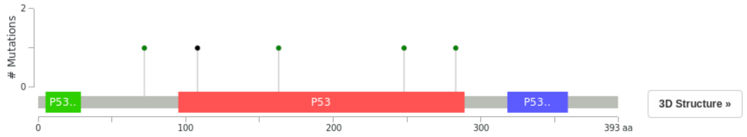

Show / hide columns | Showing 5 mutation(s) | Search:

Sample ID	AA change		Type	If Somatic	Chr	Start Pos	End Pos	Ref	Var
HCC1143	R248Q	3D	Missense	Somatic	17	7577538	7577538	G	A
HCC1954	Y163C	3D	Missense	Somatic	17	7578442	7577538	A	G
HCC1187	G108delG	3D	IF del	Somatic	17	7578442	7577538	GGT	-
HCC2218	R283C	3D	Missense	Germ-line	17	7577091	7577091	G	A
HCC2218	P72R		Missense	Germ-line	17	7579472	7579472	G	C

Supplementary Figure S9. TP53 Germ-line and somatic mutations in in each cell line. Mutations annotated by COSMIC, shows the somatic mutations including 1 missense mutation for HCC1143, 1 missense mutation for HCC1954 and 1 inframe deletion for HCC1187. The two germ-line missense mutations for HCC2218 were from aforementioned analysis. The figure is generated from MutationMapper (11). Missense Mutations are showed in green and Inframe Mutations are showed in black. The top colored bars showed the location of the mutations in TP53 as well as the domains of TP53. The table underneath shows the details about the mutation types.

References

1. Carter, S.L., Cibulskis, K., Helman, E., McKenna, A., Shen, H., Zack, T., Laird, P.W., Onofrio, R.C., Winckler, W., Weir, B.a. *et al.* (2012) Absolute quantification of somatic DNA alterations in human cancer. *Nature biotechnology*, **30**, 413-421.
2. Carter, S.L., Meyerson, M. and Getz, G. (2011) Accurate estimation of homologue-specific DNA concentration-ratios in cancer samples allows long-range haplotyping. *Scott L. Carter*, 59.
3. Van Loo, P., Nordgard, S.H., Lingjærde, O.C., Russnes, H.G., Rye, I.H., Sun, W., Weigman, V.J., Marynen, P., Zetterberg, A. and Naume, B. (2010) Allele-specific copy number analysis of tumors. *Proceedings of the National Academy of Sciences*, **107**, 16910-16915.
4. Yau, C., Mouradov, D., Jorissen, R.N., Colella, S., Mirza, G., Steers, G., Harris, A., Ragoussis, J., Sieber, O. and Holmes, C.C. (2010) A statistical approach for detecting genomic aberrations in heterogeneous tumor samples from single nucleotide polymorphism genotyping data. *Genome biology*, **11**, R92-R92.
5. Li, Y. and Xie, X. (2014) Deconvolving tumor purity and ploidy by integrating copy number alterations and loss of heterozygosity. *Bioinformatics*, **30**, 2121-2129.
6. Xi, R., Hadjipanayis, A.G., Luquette, L.J., Kim, T.-M., Lee, E., Zhang, J., Johnson, M.D., Muzny, D.M., Wheeler, D.A. and Gibbs, R.A. (2011) Copy number variation detection in whole-genome sequencing data using the

Bayesian information criterion. *Proceedings of the National Academy of Sciences*, **108**, E1128-E1136.

7. Yu, Z., Liu, Y., Shen, Y., Wang, M. and Li, A. (2014) CLImAT : accurate detection of copy number alteration and loss of heterozygosity in impure and aneuploid tumor samples using whole-genome sequencing data. *Bioinformatics*, **30**, 413-421.
8. Oesper, L., Satas, G. and Raphael, B.J. (2014) Quantifying tumor heterogeneity in whole-genome and whole-exome sequencing data. *Bioinformatics*, **30**, 3532-3540.
9. Yau, C. (2013) OncoSNP-SEQ: a statistical approach for the identification of somatic copy number alterations from next-generation sequencing of cancer genomes. *Bioinformatics*, **29**, 2482-2484.
10. Gusnanto, A., Wood, H.M., Pawitan, Y., Rabbitts, P. and Berri, S. (2012) Correcting for cancer genome size and tumour cell content enables better estimation of copy number alterations from next-generation sequence data. *Bioinformatics*, **28**, 40-47.
11. Gao, J., Aksoy, B.A., Dogrusoz, U., Dresdner, G., Gross, B., Sumer, S.O., Sun, Y., Jacobsen, A., Sinha, R., Larsson, E. *et al.* (2013) Integrative Analysis of Complex Cancer Genomics and Clinical Profiles Using the cBioPortal. *Science Signaling*, **6**, pl1-pl1.
